# Supplementary material for: Extracellular Vesicles from Lactobacillus rhamnosus BS-Pro-08, Kefir Grain, Suppress Adipogenesis and Enhance Lipolysis in Adipocytes
Source: Int J Mol Sci. 2025 Dec 4;26(23):11732. doi: 10.3390/ijms262311732 (PMC12692708; doi:10.3390/ijms262311732)
Supplement: Supplementary file 1 [file ijms-26-11732-s001.zip › ijms-3998273-supplementary.pdf]

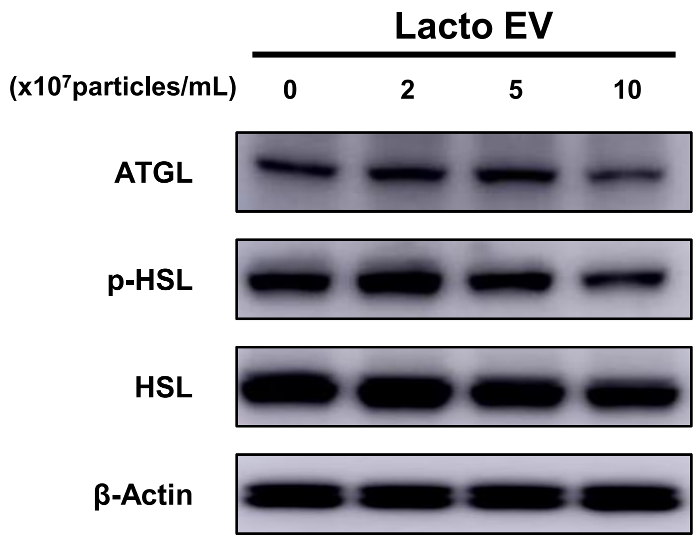

Figure S1. Western blot analysis of canonical ATGL/HSL signaling in 3T3-L1 adipocytes treated with Lacto EV.
Representative immunoblots showing the expression levels of adipose triglyceride lipase (ATGL), total hormone-sensitive lipase (HSL), and phosphorylated HSL (p-HSL) in mature 3T3-L1 adipocytes following treatment with the indicated concentrations of Lacto EV. β-Actin was used as a loading control.

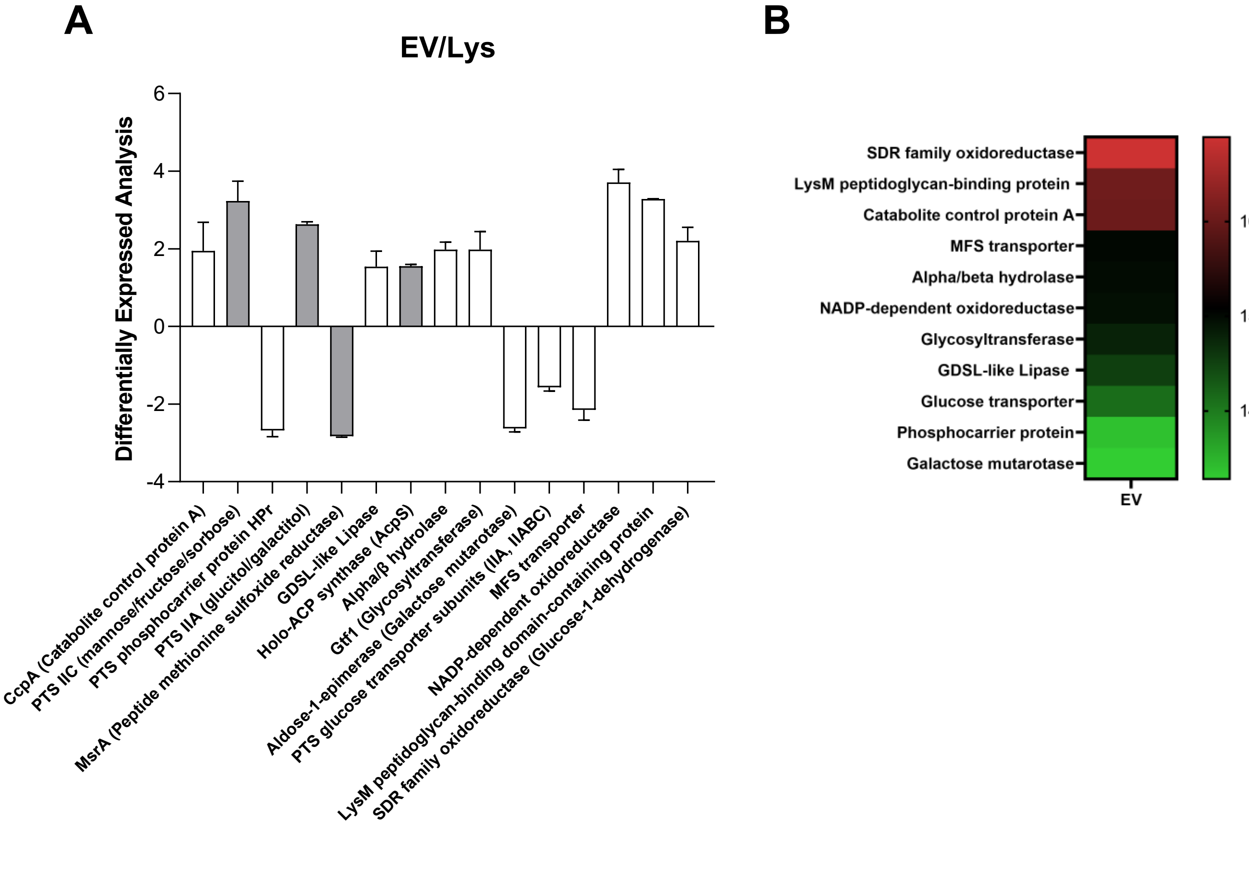

Figure S2. Proteomic comparison between Lacto EV and bacterial lysate.
Differentially expressed proteins were identified based on a fold-change cut-off value (top 95% and bottom 5%) with a significance threshold of $p < 0.05$, resulting in the identification of 151 differentially expressed proteins. Among these, 70 proteins were upregulated and 81 were downregulated in Lacto EV compared to the lysate group. From this dataset, 15 proteins with potential anti-adipogenic relevance were selected based on literature analysis. **A)** The results are presented as a differentially expressed analysis showing median log_2 fold-change (log_2FC) with 95% confidence intervals (CI). Gray bars indicate proteins exhibiting expression patterns opposite to those typically associated with adipogenesis inhibition. **B)** Heat map depicting the expression levels of the 11 anti-adipogenic candidate proteins in Lacto EV; the color scale represents relative abundance (red, higher expression; green, lower expression).
